# Epigenetic equilibrium in chromatinopathies: network instability in neurodevelopment

**DOI:** 10.3389/fneur.2026.1848929

**Published:** 2026-06-19

**Authors:** Himanshu Goel

**Affiliations:** Hunter Genetics, HNEkidshealth, Waratah, NSW, Australia

**Keywords:** chromatin load, chromatinopathies, endophenotypes, epigenetic equilibrium, epilepsy, network instability, neurodevelopmental disorders, precision neurology

## Abstract

**Background:**

Chromatin-modifying systems regulate transcriptional programs essential for human neurodevelopment through dynamic modification of histones, DNA, and higher-order chromatin architecture. Pathogenic variants affecting these systems give rise to chromatinopathies, a heterogeneous group of disorders characterised by consistent neurological features, including intellectual disability, developmental delay, autism spectrum disorder, epilepsy, and language impairment, alongside directional variation in somatic traits such as growth and skeletal development. This discordance challenges linear genotype–phenotype models.

**Methods:**

This conceptual review synthesises genetic, epigenomic, transcriptomic, cellular, neuroimaging, and electrophysiological evidence to develop an epigenetic equilibrium model. The model proposes that neurodevelopment depends on context-specific balance among activation-associated and repressive chromatin mechanisms. Deviation from this range disrupts transcriptional fidelity and neural network stability. The concepts of chromatin load, network capacity, and mirror endophenotyping are used to explain variable expressivity, direction-sensitive somatic phenotypes, and convergent neurological outcomes.

**Results:**

Despite molecular diversity, chromatinopathies converge neurologically due to disruption of transcriptional equilibrium. We introduce an epigenetic equilibrium model incorporating chromatin load, network capacity, and transcriptional dynamics. We further define mirror endophenotyping as a framework capturing reciprocal directionality of intermediate phenotypes across shared chromatin axes.

**Conclusion:**

Chromatinopathies are best understood as systems-level disorders of transcriptional regulation rather than isolated molecular defects. This framework provides a unifying mechanistic explanation for phenotypic convergence across chromatinopathies and introduces a systems-level approach to diagnosis and therapy. This approach provides a foundation for precision neurology in neurodevelopmental disease.

## 1 Introduction

Human neurodevelopment requires the precise coordination of gene expression across spatial and temporal dimensions. The genome provides the sequence-based blueprint, but chromatin determines how and when this blueprint is executed. This distinction is particularly important in the nervous system, where developmental processes are prolonged, tightly staged, and highly sensitive to perturbation. The central nervous system is assembled through a sequence of tightly regulated processes that include neural induction, progenitor proliferation, lineage commitment, neuronal migration, axon guidance, dendritic arborisation, synaptogenesis, myelination, and activity-dependent refinement of neuronal circuits. These processes must occur in a coordinated and ordered fashion. Genes relevant at one developmental stage must be activated at the appropriate time, silenced when no longer required, and often reactivated in a different context later in development. The result is not merely production of neurons, but assembly of highly ordered and functionally specialised circuits capable of supporting cognition, language, motor control, memory, and adaptive behaviour (1, 2).

Chromatin architecture regulates transcription through multiple interacting mechanisms, including histone modifications, DNA methylation, nucleosome positioning, enhancer–promoter interactions, and three-dimensional genome organisation (3, 4). These layers function together to control transcriptional accessibility, timing, and coordination. Neural development depends on this regulatory precision, as processes such as progenitor proliferation, neuronal differentiation, migration, synaptogenesis, and activity-dependent plasticity all require tightly orchestrated transcriptional programs. DNA is packaged into nucleosomes composed of histone proteins, and chromatin configuration determines whether specific genomic regions are permissive or refractory to transcription. In this sense, chromatin is not a passive scaffold but an active and continuously remodelled regulatory interface between the genome and cellular phenotype (5–7).

Over the past decade, genomic studies have identified a substantial proportion of neurodevelopmental disorders arising from variants in genes encoding chromatin regulators (8, 9). These include not only histone-modifying enzymes, but also chromatin readers, nucleosome remodellers, architectural proteins, and DNA methylation machinery. These disorders, which are increasingly grouped under the umbrella term chromatinopathies, include conditions such as Kabuki syndrome, Weaver syndrome, Sotos syndrome, Rubinstein–Taybi syndrome, Wiedemann–Steiner syndrome, Koolen-de Vries syndrome, and multiple X-linked intellectual disability syndromes (10–15). Although each condition has its own molecular basis and characteristic features, the shared neurological phenotype is striking. Developmental delay, intellectual disability, language dysfunction, autism spectrum disorder, epilepsy, hypotonia, behavioural dysregulation, and learning difficulties are observed across many chromatinopathies. This overlap occurs even though the affected genes may encode writers, erasers, readers, remodelers, architectural proteins, or DNA methylation regulators, and even though some of these proteins act on chromatin marks with apparently opposite transcriptional effects. The convergence of neurological features, therefore, does not imply that the primary molecular defects are identical. Rather, different primary chromatin defects can converge on a limited set of vulnerable neurodevelopmental processes, including cortical patterning, neuronal differentiation, synaptic maturation, activity-dependent transcription, and circuit excitability. Supporting this systems-level interpretation, several chromatinopathies are associated with reproducible DNA methylation episignatures, indicating that primary genetic defects in epigenetic regulators can generate measurable downstream epigenomic states that may assist diagnosis and variant interpretation (16, 17). Representative chromatin regulators implicated in neurodevelopmental disorders, their principal epigenetic functions, and associated somatic growth tendencies are summarised in Table 1.

**Table 1:** Representative chromatin regulators implicated in neurodevelopmental disorders and why a linear model is insufficient.

Regulator/syndrome	Principal chromatin function	Direction-sensitive somatic feature	Implication for the equilibrium model
EZH2/Weaver syndrome; PRC2-related overgrowth	PRC2 H3K27 methyltransferase; establishes a repressive H3K27me3-associated state.	Overgrowth and advanced skeletal maturation are typical, consistent with reduced repressive capacity in growth-relevant tissues (9, 62).	Loss of a repressive writer can increase derepression while still producing NDD phenotypes; “reduced activity” does not mean reduced transcriptional output.
KDM6A/Kabuki syndrome type 2	H3K27me3 demethylase; removes a repressive mark and contributes to enhancer regulation.	Growth deficiency/short stature; mouse models show reduced bone length and growth-plate defects (63, 64).	Impaired removal of repression can generate a somatic phenotype opposite to PRC2-related overgrowth, while converging neurologically.
KMT2D/Kabuki syndrome type 1	COMPASS-family H3K4 methyltransferase, especially enhancer-associated H3K4me1, with broader context-dependent functions.	Postnatal growth deficiency, skeletal anomalies, and craniofacial patterning defects (8, 65).	An activation-associated writer can cause NDD through enhancer dysregulation; not simply opposite to KDM5C at the same genomic compartment.
KMT2C/KMT2C-related NDD	COMPASS-family enhancer-associated H3K4 methyltransferase with partial functional overlap with KMT2D.	Recent cohort data support short stature and a phenotype distinct from Kleefstra and Kabuki syndromes (42).	KMT2C and KMT2D illustrate partial molecular overlap but non-identical network engagement, explaining phenotypic divergence plus NDD convergence.
KMT2A/Wiedemann-Steiner syndrome	H3K4 methyltransferase acting at promoters and regulatory regions; activation-associated chromatin function.	Short stature, hypertrichosis, facial gestalt, and variable growth effects (66).	Provides a more direct H3K4 writer comparator for KDM5C than KMT2D in some experimental models (49).
KDM5C/Claes-Jensen syndrome and X-linked ID	H3K4me2/3 demethylase enriched at promoters, with context-dependent effects on neuronal transcription and enhancers.	Variable growth and head-size features; core phenotype is ID, seizures, and behavioural disturbance (41).	Shows that erasers may maintain transcriptional precision rather than simply suppress activation globally.
NSD1/Sotos syndrome	H3K36 methyltransferase linked to transcriptional elongation and gene-body chromatin states.	Overgrowth and macrocephaly are characteristic (43).	Same mark class as NSD2, but different locus/cell-type engagement produces different somatic readouts.
NSD2/NSD2-related developmental disorder	H3K36 methyltransferase; altered methylation activity affects developmental transcriptional programs.	Developmental disorder with growth deficiency or distinct growth pattern compared with NSD1-related overgrowth (44, 67).	Demonstrates that same enzymatic class does not guarantee same growth phenotype; topology and timing matter.
CREBBP/EP300 /Rubinstein-Taybi syndrome	Histone acetyltransferase and transcriptional co-activator function; H3K27ac and enhancer activation.	Growth restriction, microcephaly, broad thumbs/toes, and skeletal differences (68).	Loss of activation-associated acetylation can converge neurologically with disorders of repressive machinery.
DNMT3A / Tatton-Brown-Rahman syndrome	*De novo* DNA methyltransferase; establishes DNA methylation patterns and regulatory stability.	Overgrowth and intellectual disability are typical (69).	DNA methylation regulators demonstrate that equilibrium extends beyond histone marks to stable epigenomic state control.
CHD8 / CHD8-related NDD	ATP-dependent chromatin remodeler regulating accessibility and transcriptional networks.	Macrocephaly/overgrowth tendency with autism and developmental delay (70).	Chromatin remodeling can alter excitatory and inhibitory developmental trajectories, connecting chromatin balance to circuit imbalance (52).

ID, intellectual disability; NDD, neurodevelopmental disorder; PRC2, Polycomb repressive complex 2. The table is intended to illustrate directionality and network logic, not to provide a complete nosology of chromatinopathies.

This convergence presents a conceptual challenge. Traditional genotype–phenotype reasoning often assumes a relatively linear relationship between biochemical function and clinical outcome. If one enzyme writes an activation-associated mark and another removes it, a simple linear model would predict reciprocal phenotypes. However, chromatinopathies do not behave as isolated biochemical titrations. Different mutations within a shared regulatory pathway can produce opposite or direction-sensitive somatic traits, such as growth restriction versus overgrowth, while still converging on developmental delay, cognitive impairment, epilepsy susceptibility and behavioural abnormalities. This pattern suggests that somatic systems may often preserve directional readout of regulatory imbalance, whereas neural systems are especially sensitive to loss of regulatory precision, timing and network synchrony. Neurodevelopment may therefore be destabilised by deviation from balance itself, not only by the biochemical direction of the deviation.

This review proposes epigenetic equilibrium model for chromatinopathies. It examines how disruption of chromatin writers, erasers, readers, remodellers, architectural proteins and DNA methylation machinery can produce direction-sensitive somatic phenotypes while converging on neurodevelopmental network instability. The framework integrates chromatin load, network capacity, and mirror endophenotyping with diagnostic and therapeutic implications. t.

## 2 Chromatin regulation as a dynamical system in neurodevelopment

A useful starting point is to move away from the simplistic notion of chromatin as a static binary switch between on and off states. Chromatin regulation operates not as a binary switch but as a dynamic, multi-layered system controlling transcriptional probability, amplitude, timing, and coordination. Histone modifications, DNA methylation, nucleosome positioning, and higher-order genome architecture collectively determine regulatory accessibility and responsiveness. These layers interact non-linearly, forming complex regulatory networks in which perturbations propagate across multiple levels.

Histone methylation illustrates this principle well. Methylation of different lysine residues can have activating or repressive effects, depending on residue context, degree of methylation, interacting proteins, and genomic location. H3K4 methylation is generally associated with active promoters and poised developmental enhancers. H3K27 methylation is strongly associated with repression and Polycomb-mediated silencing (18). H3K36 methylation is linked to transcriptional elongation, enhancer regulation, and control of spurious intragenic transcription (19). These marks are established and removed by opposing enzymatic systems, but the biological consequence of changing a specific mark is not merely local. Through transcription factor recruitment, chromatin looping, enhancer function, and crosstalk with other marks, a change at one regulatory node may alter broader transcriptional states. Polycomb repressive complex (PRC2)-related overgrowth and Rubinstein-Taybi syndrome (RSTS) are, respectively, associated with impaired H3K27 methylation and acetylation. Whereas these syndromes share commonalities like intellectual disability and susceptibility to cancers, they are generally divergent in their skeletal growth phenotypes, potentially through dysregulation of their opposing H3K27 writer functions (20).

Histone acetylation provides an additional layer of regulation that is particularly important in activity-dependent transcription. Acetylation of lysine residues neutralises positive charge and generally promotes more open chromatin states, facilitating transcription factor binding and enhancer-promoter communication. In neurons, histone acetylation is closely tied to synaptic activity, memory-related transcription, and plasticity-related gene expression. The nervous system, therefore, depends not only on baseline chromatin organisation during early development but also on the capacity to remodel chromatin in response to experience dynamically (3, 5–7, 13, 21).

These mechanisms are highly interconnected. Histone methylation and acetylation do not function as separate pathways but as a densely interactive regulatory network (3). H3K27 methylation and H3K27 acetylation, for example, are mutually exclusive at the same residue and therefore function as a regulatory switch between repressed and active enhancer states. Histone modifications influence nucleosome positioning, transcription factor occupancy, and recruitment of ATP-dependent chromatin remodellers. Transcription itself feeds back into chromatin state. A perturbation in one component can therefore alter the dynamic behaviour of the whole system.

Beyond histone modifications, chromatin regulation involves multiple additional components. Chromatin readers interpret histone marks and recruit transcriptional machinery. ATP-dependent chromatin remodellers reposition nucleosomes and regulate accessibility. Architectural proteins such as cohesin and CTCF organise three-dimensional genome structure, enabling long-range interactions between enhancers and promoters (22–24). DNA methylation establishes more stable regulatory states, while non-coding RNAs contribute to locus-specific regulation (25–27). A fundamental constraint on gene regulation arises from the limited intrinsic specificity of transcription factors (TFs). Protein–DNA interactions are inherently degenerate, leading to pervasive low-affinity, non-target binding across the genome. At scale, such interactions introduce regulatory crosstalk that degrades transcriptional precision. Recent systems-level modelling demonstrates that chromatin provides a solution to this constraint by acting as an error-suppressing regulatory layer. By modulating DNA accessibility, chromatin selectively restricts TF binding to appropriate genomic regions, thereby reducing non-target interactions and improving the fidelity of gene expression programs. Importantly, this function is not purely local but operates at a global regulatory level, where chromatin reduces cumulative gene expression error across entire networks.

This systems perspective helps explain why the consequences of chromatin disruption are often non-linear. Small perturbations in highly connected nodes may have large downstream effects, while larger perturbations in buffered regions may have minimal phenotypic consequences. Similarly, reduced activity of a given enzyme does not automatically produce the opposite phenotype of increased activity in another enzyme, because the outcome depends on the mark, genomic context, cell type, developmental timing, and topology of the affected network. For example, reduced activity of an activation-associated writer may lower transcriptional competence at selected enhancers or promoters, whereas reduced activity of a repressive writer, such as a PRC2 component may cause derepression of normally silenced programs. Both are directionally different, but either can push neural gene regulation outside the tolerated range. Neurons are uniquely dependent on this regulatory precision. In addition to developmental programs, they must maintain activity-dependent transcriptional responses required for plasticity and circuit refinement. Chromatin, therefore, integrates developmental and experience-dependent signals, making the nervous system particularly vulnerable to dysregulation. Chromatin does not simply enable transcription—it constrains regulatory entropy, allowing complex gene expression programs to be executed reliably despite biophysical limitations of TF specificity (28).

## 3 Chromatin regulation: neural circuits and activity-dependent neuronal pruning

Neural circuit formation depends on tightly regulated transcriptional programs that guide synaptogenesis, maturation, and activity-dependent pruning. During development, an excess of synapses is initially formed, followed by selective elimination through pruning mechanisms guided by neuronal activity and transcriptional programs. Neural stem and progenitor cells must maintain multipotency early in development while retaining the capacity to commit to highly specific lineages later. Chromatin-mediated regulation is particularly suited to this requirement because it separates binary and analogue control. At a first layer, chromatin accessibility determines whether genes are permissive or non-permissive (ON/OFF gating). At a second layer, TFs modulate expression levels within accessible regions. This two-tier architecture ensures that inappropriate activation of silent programs is minimised while allowing graded control of active genes. Failure of this architecture leads to predictable circuit-level consequences. Loss of chromatin-mediated repression may permit inappropriate activation of immature or non-lineage programs, contributing to persistence of immature synaptic connections and impaired pruning. Conversely, insufficient activation-associated chromatin function may prevent the timely induction of maturation, cytoskeletal, synaptic, or activity-dependent genes. Excessive permissive activity or impaired boundary maintenance may also increase transcriptional noise and aberrant pruning. These mechanisms are not equivalent at the molecular level, but all can destabilise circuit refinement when they disrupt the temporal precision of neural gene expression.

Cortical development is especially sensitive to these processes. Radial glial cells generate deep-layer neurons first, followed by upper-layer projection neurons, with each stage depending on chromatin-regulated transcriptional programs that specify laminar identity. Perturbation of these programs can affect cortical thickness, areal patterning, neuronal density, and the ratio of excitatory neuronal subtypes. Even when gross malformations are absent, subtle alterations in laminar architecture or neuronal subtype composition may have important consequences for cortical connectivity and cognition (29).

Migration represents a second crucial stage. Newly born neurons must interpret extracellular cues, reorganise cytoskeletal structure, and move into appropriate cortical positions. Chromatin regulation influences the expression of adhesion molecules, cytoskeletal regulators, and guidance receptor systems required for this process. Disruption may result in overt migration defects or more subtle disturbances in neuronal positioning that alter microcircuit organisation. Such changes may not always be visible on conventional imaging but can nevertheless influence excitability, synchrony, and information processing (29).

Axon guidance and target selection provide a third stage in which chromatin perturbation may have amplifying consequences. Long-range connectivity depends on precise temporal regulation of guidance molecules, receptor expression, and downstream signalling effectors. An early disturbance in the transcriptional regulation of these systems may alter the topology of brain networks in enduring ways. The same applies to dendritic arborisation and spine development, which require coordinated expression of structural proteins, synaptic scaffolds, and local translational machinery. Multiple chromatinopathies exhibit abnormalities in dendritic morphology or synaptic development in experimental systems, reinforcing the idea that chromatin regulators act upstream of core circuit-building programs.

Insufficient activation-associated chromatin function may impair induction of maturation and pruning programs, resulting in hyperconnected but inefficient networks. By contrast, impaired repression, derepression, or excessive permissive activity may destabilise regulatory boundaries and promote inappropriate pruning or loss of synaptic specificity, leading to hypoconnected circuits (30). Despite these different proximal routes, both states can result in network instability. The combination of structurally altered circuitry and reduced plastic buffering may explain why chromatinopathies so frequently produce both developmental delay and later neuropsychiatric complications.

## 4 Neurological convergence across chromatinopathies

The recurrent neurological phenotype seen in chromatinopathies is one of the strongest arguments for a systems-level interpretation. Intellectual disability is common, but the cognitive profile often extends beyond global developmental delay. Language acquisition is particularly vulnerable. Speech may be delayed, expressive language disproportionately impaired, or verbal learning and pragmatic communication selectively affected (31, 32). Executive dysfunction, attentional abnormalities, emotional dysregulation, and autism-related social communication difficulties are also frequent. Such recurrence across disorders affecting different chromatin regulators implies convergence on common cortical and subcortical networks rather than isolated gene-specific effects. The same applies to behavioural and psychiatric comorbidity. Autism spectrum disorder, ADHD-like symptoms, anxiety, mood dysregulation, aggression, self-injury, and sleep disturbance recur across many chromatin disorders (33). These features likely reflect altered maturation and modulation of distributed networks involved in salience processing, executive control, social cognition, and emotional regulation. Again, what is notable is not phenotypic identity, but recurring network-level themes across genetically distinct disorders.

Hypotonia is another recurring feature and likely reflects multi-level dysfunction involving motor planning, corticospinal pathways, cerebellar function, and neuromuscular coordination. Feeding difficulties, oral-motor problems, and delayed gross motor milestones are common in infancy. This broad overlap suggests that chromatin imbalance affects both cognitive and sensorimotor developmental programs (34, 35).

Epilepsy strengthens the case for convergent circuit vulnerability. Some chromatinopathies are strongly epilepsy-associated, while in others seizures are variable or less frequent, but the overall burden is greater than would be expected by chance. Importantly, epilepsy phenotypes are heterogeneous. Some patients have focal epilepsies, others generalised epilepsies or developmental and epileptic encephalopathies, and some show abnormal EEG patterns without overt seizures. This diversity suggests that chromatin imbalance does not map onto a single epilepsy syndrome but instead increases network fragility in a way that interacts with brain region, developmental timing, and individual modifiers (36, 37).

Neuroimaging findings are likewise convergent but non-specific. Cortical volume differences, white matter abnormalities, corpus callosum anomalies, cerebellar differences, delayed myelination, and hippocampal changes have all been reported across different chromatinopathies. None is pathognomonic, yet together they point to widespread effects on neurodevelopmental architecture. This pattern is compatible with a model in which chromatin perturbation acts upstream of multiple circuit-building pathways rather than producing one stereotyped structural lesion (1, 38–40).

## 5 Somatic polarity and mirrored phenotypes

Comparative analysis of chromatin disorders reveals a consistent pattern of phenotypic mirroring. Disorders affecting opposing regulators of the same histone residue frequently exhibit polarity in somatic traits while maintaining overlapping neurodevelopmental features. Growth may be increased or decreased, head circumference may trend toward macrocephaly or microcephaly, and skeletal maturation may be advanced in some conditions and delayed in others. At first glance, these mirrored somatic features appear to contradict the shared neurodevelopmental phenotype. However, this apparent inconsistency provides a critical insight into how chromatin regulation operates across different biological systems.

The clearest examples arise from regulators that act on shared chromatin axes, although they do not always oppose each other at the same genomic element. Perturbation of H3K27 methylation illustrates true directional polarity: reduced EZH2/PRC2 methyltransferase activity decreases a repressive H3K27me3-associated function and is associated with Weaver overgrowth, whereas impaired KDM6A demethylase function limits removal of H3K27me3 and is associated with Kabuki syndrome type 2 and growth deficiency. This pair, therefore, provides a useful example of how altered repression can generate direction-sensitive somatic consequences while preserving neurological convergence (8, 9, 41). The H3K4 axis requires more careful interpretation. KMT2D and KMT2C are enhancer-associated H3K4 methyltransferases, whereas KDM5C primarily demethylates promoter-enriched H3K4me2/3 and has additional context-dependent neuronal functions (42). KMT2D and KDM5C, therefore, should not be presented as a simple writer–eraser pair acting on the same residue at the same genomic compartment. A more direct experimental example of H3K4 balance is the KMT2A-KDM5C relationship, in which combined perturbation in model systems partially corrected behavioural, dendritic, transcriptomic, and H3K4 methylation abnormalities. This supports the equilibrium concept, but also emphasises that the relevant axis is defined by genomic context, cell type, and network output, not merely by the name of the histone residue.

The KMT2C-KMT2D relationship further illustrates why a linear model is insufficient. Both genes encode COMPASS-family enhancer-associated H3K4 methyltransferases, yet *KMT2D* pathogenic variants classically cause Kabuki syndrome, whereas recent large cohort data indicate that KMT2C-related neurodevelopmental disorder is clinically and epigenetically distinct from both Kleefstra and Kabuki syndromes (42). KMT2C-related disease appears particularly enriched for developmental delay, intellectual disability, behavioural and psychiatric problems, hypotonia, seizures, and short stature. These differences likely reflect non-identical enhancer occupancy, developmental timing, complex membership, dosage sensitivity, and tissue-specific network engagement. In the equilibrium model, KMT2C and KMT2D therefore fit not as interchangeable enzymes but as partially overlapping regulators whose perturbation can affect different somatic modules while still converging on fragile neurodevelopmental networks. Variation within a single enzymatic class reinforces this point. NSD1 and NSD2 are both H3K36 methyltransferases, yet loss of function in these genes is associated with divergent growth outcomes (27–29). This cannot be explained by simple biochemical opposition, but it does fit a network model in which the phenotypic result depends on the specific loci, developmental stages, and circuits most affected (43–45).

## 6 The epigenetic equilibrium model

The epigenetic equilibrium model, in its simplest form, proposes that neurodevelopment depends on maintaining chromatin regulatory output within a constrained functional range. This range is not defined by a single absolute level of a histone mark, nor by a generic quantity termed chromatin activity. It is defined by context-specific balance among activation-associated marks, repressive marks, chromatin accessibility, writer and eraser activity, transcriptional responsiveness, and network stability. Within this range, transcriptional programs are sufficiently stable to preserve cellular identity yet sufficiently flexible to respond to developmental and activity-dependent cues. Outside this range, transcriptional homeostasis is lost. This concept can be formalised using insights from recent systems biology models of gene regulation. These models define a measurable quantity, global gene expression error (GEE), which captures the deviation between desired and actual transcriptional outputs across gene networks. Chromatin-based regulation minimises this error by suppressing inappropriate activation of genes that should remain silent and by improving the dynamic range of expression for active genes.

**Figure 1:**
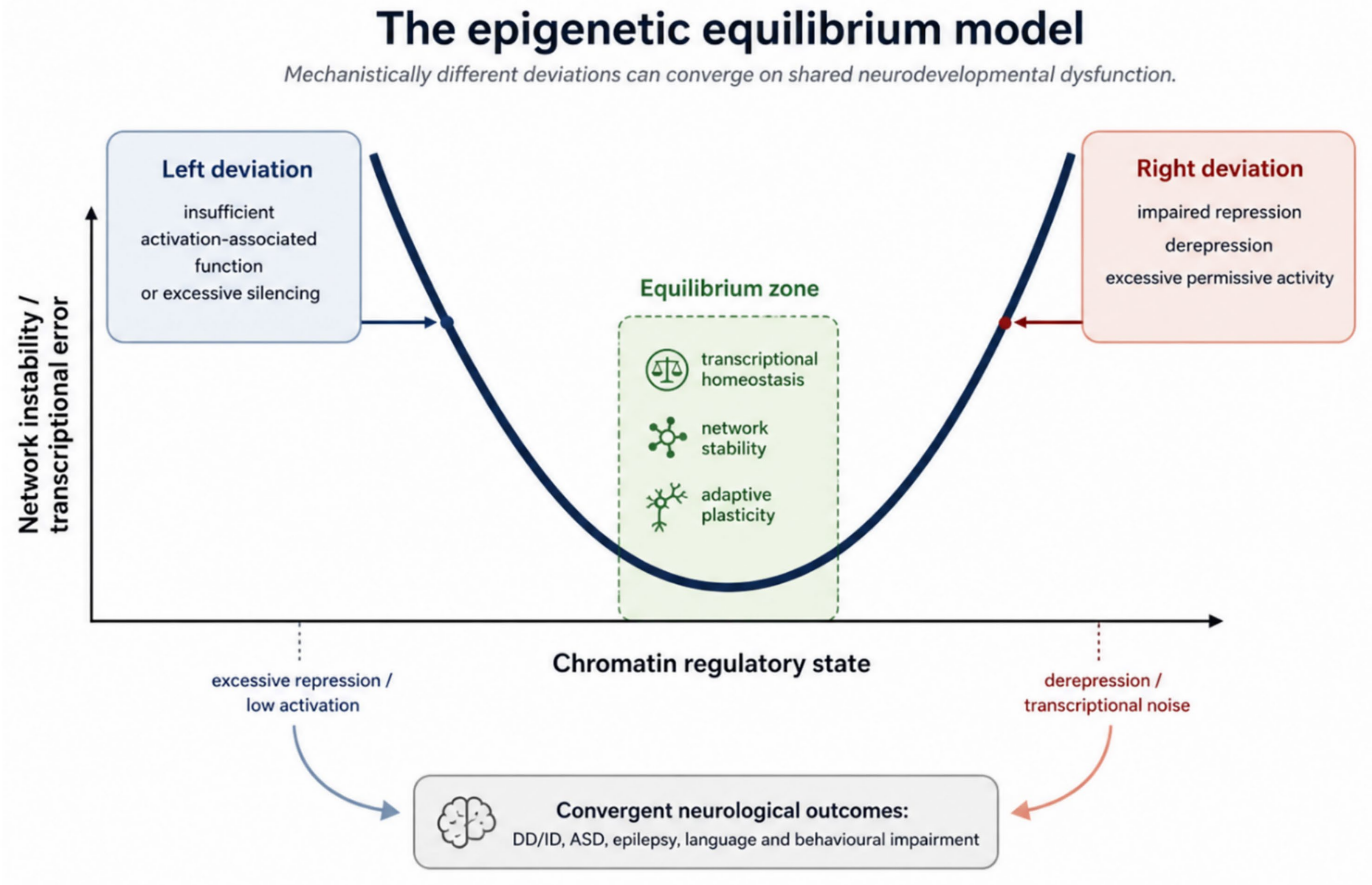
The epigenetic equilibrium model. Network instability or transcriptional error increases when chromatin regulatory state moves outside a context-specific equilibrium range. Leftward deviations may reflect insufficient activation-associated function or excessive silencing; rightward deviations may reflect impaired repression, derepression, transcriptional noise. Both routes can converge on shared neurological outcomes.

The relationship between chromatin regulatory output and developmental outcome is non-linear. Both leftward and rightward deviations from the tolerated range impair function, producing a U-shaped relationship when the *y*-axis is conceptualised as transcriptional error or network instability. A leftward deviation may reflect insufficient activation-associated function, excessive repression, delayed lineage transitions, or persistence of inappropriate silencing. A rightward deviation may reflect impaired repression, derepression, disrupted regulatory boundaries, transcriptional noise, or ectopic activation. These states are mechanistically different, but both can destabilise shared neural networks through loss of transcriptional fidelity and regulatory coordination. This model explains why disorders affecting biochemically opposite enzymes can converge neurologically without requiring their molecular effects to be identical. It also accommodates partial compensation. A system pushed modestly away from equilibrium may remain clinically compensated if buffering mechanisms are intact, whereas a second perturbation, developmental stress, or environmental insult may push it beyond threshold and unmask disease. The equilibrium model, therefore, naturally connects molecular perturbation with variable penetrance and fluctuating clinical expression (Figure 1).

Another important feature is that equilibrium is context-dependent. Different cell types, brain regions, and developmental stages may have different optimal ranges and different tolerances for deviation. Deep-layer corticocortical projection neurons may be vulnerable at one developmental stage, while interneuron maturation or cerebellar circuitry may be vulnerable later. This means that equilibrium should not be imagined as a single universal point, but as a family of context-specific functional ranges across the developing nervous system.

## 7 Chromatin network fragility

A systems model of chromatinopathies must explain why genome-wide or broad regulatory perturbation produces relatively stereotyped clinical phenotypes rather than completely nonspecific dysfunction. The concept of chromatin network fragility addresses this problem. The central idea is that chromatin modifiers act broadly, but only certain nodes within gene regulatory networks are both highly connected and highly sensitive to chromatin imbalance. Disruption of these fragile hubs leads to cascading effects across the network, amplifying initial perturbations and producing system-level dysfunction (17).

Fragile nodes are not single genes in a fixed list; they are network positions where small expression changes can alter developmental trajectory or circuit function. Examples include transcription factors controlling cortical lineage transitions and laminar identity; Polycomb-regulated developmental signalling pathways such as WNT, SHH, BMP/TGF-beta, Notch, and retinoic acid signalling (46); synaptic scaffolding and adhesion modules; activity-regulated immediate early gene programs; cytoskeletal and axon-guidance pathways; interneuron specification and maturation programs; ion-channel and neurotransmitter receptor networks; mitochondrial and metabolic resilience pathways (47); and myelination or glial maturation modules (48). If chromatin perturbation selectively increases variability or reduces precision at such hubs, the resulting network effect can be much greater than would be predicted by average genome-wide change. Conversely, perturbation of genes that are buffered, redundant, or peripherally connected may produce little phenotypic signal.

This concept has several implications. First, it explains phenotypic convergence: different chromatin regulators may perturb different immediate targets, but if those perturbations converge on the same fragile network hubs, the clinical phenotype will overlap (49, 50). Second, it explains why some chromatin disorders have strong epilepsy associations: if vulnerable nodes lie in excitability, synaptic inhibition, or activity-responsive transcriptional pathways, network instability may emerge as seizures (48). Third, it explains selective tissue vulnerability. The same chromatin perturbation may be tolerated in one tissue but not another, depending on whether fragile nodes are engaged and how much redundancy exists.

Chromatin network fragility also suggests an important distinction between measurable molecular change and pathogenic molecular change. Not every transcriptomic or epigenomic abnormality contributes equally to disease. Some changes may represent buffered background noise, while others alter critical network hubs. A major task for future work will be to identify which molecular signatures correlate most strongly with clinically meaningful fragility.

## 8 Chromatin load and network capacity

Chromatin load refers to the cumulative burden of transcriptional dysregulation imposed on regulatory networks by a chromatin perturbation. Rather than focusing on single genes or single histone marks, this concept captures the distributed effect of dysregulated chromatin across multiple loci, pathways, and developmental stages.

Chromatin load can be understood as comprising several components. One component is magnitude: how strongly are transcriptional states perturbed? A second is breadth: how many relevant loci or pathways are affected? A third is timing: at what developmental stage does the perturbation occur? A fourth is persistence: does the perturbation resolve, fluctuate, or continue over time? A fifth is topological weighting: does the perturbation fall primarily on buffered loci or on fragile network hubs? Together, these variables determine the effective burden that a chromatin perturbation places on a developing neural system.

Opposing this burden is network capacity, the intrinsic ability of a neural system to absorb, buffer, and compensate for perturbation without losing functional integrity. Network capacity depends on genetic background, redundancy of pathways, developmental plasticity, metabolic resilience, synaptic adaptability, and perhaps environmental influences such as early intervention, stimulation, or stress exposure. Systems with high capacity may tolerate substantial chromatin load before clinical symptoms emerge. Systems with low capacity may decompensate under relatively modest load.

The relationship between chromatin load and network capacity provides a useful framework for understanding variable expressivity and incomplete penetrance. Two individuals with the same pathogenic variant may not share the same phenotype because their network capacity differs. One may remain relatively compensated, while the other develops severe epilepsy or profound intellectual disability. Similarly, some individuals may cross symptomatic thresholds only later in development, when cumulative load rises or compensatory mechanisms become exhausted.

This framework also helps explain episodic decompensation. A child with a chromatinopathy may appear relatively stable, then deteriorate with seizure onset, developmental regression, sleep disruption, hormonal change, or illness. Such events may transiently increase effective load or decrease available network capacity, exposing pre-existing fragility. In this way, chromatinopathies can be understood not only as static developmental disorders but as conditions with shifting system dynamics across time.

From a neurological viewpoint, chromatin load provides a bridge between molecular dysregulation and clinical phenotype. EEG slowing, epileptiform discharges, loss of developmental trajectory, behavioural regression, and network-level imaging abnormalities can all be interpreted as manifestations of a system approaching or exceeding its buffering capacity. This offers a more nuanced interpretation than simple genotype-to-symptom mapping.

The equilibrium model is compatible with established neurodevelopmental mechanisms such as excitation-inhibition imbalance, synaptic dysfunction, altered neuronal maturation, and abnormal circuit synchronisation. These mechanisms should not be considered alternatives to chromatin imbalance; rather, they are downstream network states through which chromatin dysregulation can become clinically visible. Chromatin regulators influence the timing and proportion of excitatory and inhibitory lineage specification, expression of ion channels and neurotransmitter receptors, synaptic scaffolding, axon guidance, dendritic maturation, and activity-dependent transcription. Perturbation of these programs can shift cortical and subcortical circuits toward excessive excitation, insufficient inhibition, impaired homeostatic plasticity, or unstable oscillatory synchronisation (48, 51).

In this sense, excitation-inhibition imbalance represents a circuit-level endophenotype of chromatin disequilibrium. A child with a chromatinopathy may develop epilepsy, abnormal EEG background organisation, autism-related sensory dysregulation, or attentional instability, not because the chromatin regulator is an ion-channel gene, but because disrupted transcriptional timing alters the development and adaptive calibration of excitatory and inhibitory networks. The same logic applies to other recurrent NDD mechanisms: synaptic dysfunction, abnormal pruning, impaired myelination, altered mitochondrial resilience, and disrupted activity-dependent plasticity can all be interpreted as downstream expressions of increased chromatin load acting on fragile neurodevelopmental nodes.

## 9 Neurological diagnostic implications

A major clinical value of this framework is that it offers a way to move beyond purely gene-centric diagnosis. Current diagnostic practice in genomic medicine often identifies a variant and then asks whether that specific gene explains the phenotype. While this remains essential, it can be limiting in chromatinopathies because many relevant disorders converge neurologically despite molecular diversity. A chromatin-based diagnostic framework instead asks how a variant perturbs regulatory balance, what direction of imbalance it implies, which network domains are likely to be affected, and whether the neurological phenotype is compatible with a state of elevated chromatin load in vulnerable systems.

This approach has several practical implications. First, it improves the interpretation of variants of uncertain significance. A rare variant in a chromatin regulator may be difficult to classify on sequence data alone. However, if the patient shows a neurodevelopmental phenotype strongly consistent with chromatin network dysfunction, supported by behavioural profile, growth pattern, craniofacial gestalt, EEG abnormalities, and perhaps methylation signature, the overall case for pathogenicity may be strengthened. Conversely, a variant in a chromatin regulator found in a patient whose phenotype lacks any network-level coherence may warrant greater caution.

Second, the model allows diagnostic stratification by regulatory direction rather than by gene alone. Disorders can be conceptually grouped into hypoactive chromatin states, hyperactive chromatin states, and repressive imbalance states, recognising that such categories are simplifications. These groupings may help predict mirrored somatic features, likely network vulnerabilities, and perhaps response to targeted intervention.

Third, the framework encourages systematic use of endophenotypes. In neurology, useful endophenotypes are measurable intermediate phenotypes that connect molecular lesion to clinical presentation. For chromatinopathies, these may include EEG background organisation, epileptiform burden, sleep architecture, MRI-derived cortical thickness or white matter connectivity, language profile, motor coordination phenotype, or disorder-specific methylation episignatures. These measures may be particularly valuable when genomic interpretation is ambiguous.

Fourth, the framework suggests that neurological diagnosis should explicitly incorporate developmental timing. The same chromatin perturbation may present differently in infancy, childhood, adolescence, or adulthood. Early hypotonia and feeding difficulties may later evolve into language impairment and ADHD-like symptoms, then into executive dysfunction or epilepsy. Diagnosis is therefore best understood as dynamic profiling of a chromatin-perturbed nervous system rather than one-time attribution of a fixed syndrome label.

Finally, the model supports a network-based view of differential diagnosis. Chromatinopathies overlap clinically with synaptopathies, transcription factor disorders, DNA repair syndromes, and broader developmental epilepsies. A systems-neurology perspective may help identify when a patient’s phenotype is more likely to reflect core circuit-building dysfunction, excitability network instability, or a mixed regulatory disorder.

## 10 Multi-omic and neurobiological endophenotypes

Advances in multi-omic technologies provide measurable readouts of chromatin dysregulation. DNA methylation episignatures have demonstrated clinical utility in diagnosing chromatinopathies and classifying variants of uncertain significance (43, 52, 53). Representative studies investigating molecular, multi-omic, and neurobiological endophenotypes in chromatinopathies and related neurodevelopmental disorders are summarised in Table 2.

**Table 2:** Representative multi-omic and neurobiological endophenotype studies relevant to chromatinopathies.

Study/domain	Assay/model	Endophenotype or readout	Contribution to the equilibrium model
Genome-wide DNA methylation signatures in Mendelian disorders	Peripheral blood DNA methylation arrays and machine-learning classifiers.	Syndrome-specific episignatures; VUS classification; detection of mosaicism and episignature overlap (71).	Provides a clinically scalable downstream molecular readout of chromatin disequilibrium.
PRC2/EZH2-related overgrowth	Genome-wide DNA methylation in EZH2, EED, and SUZ12-related overgrowth cohorts.	A PRC2-related methylation signature that classifies variants and captures overgrowth/intellectual disability biology (72).	Links a repressive chromatin complex to measurable methylomic state and directional growth phenotype.
KMT2C-related neurodevelopmental disorder	Large clinical cohort with phenotyping and DNA methylation analysis.	Developmental delay, ID, behavioural/psychiatric features, seizures, short stature, and a DNAm profile distinct from Kleefstra and Kabuki syndromes (42).	Shows that related enhancer writers may produce distinct episignatures while converging neurologically.
Autism and NDD transcriptomic convergence	Post-mortem brain transcriptomics and systems-level gene co-expression analyses.	Shared dysregulation of synaptic, neuronal differentiation, immune/glial, and activity-dependent modules (73).	Supports convergence from diverse genetic lesions onto common developmental and synaptic networks.
Pooled perturbation of NDD risk genes	CRISPR perturbation of NDD genes in iPSC-derived neural progenitors, glutamatergic neurons, GABAergic neurons, and zebrafish models.	Cell-type-specific convergent transcriptional networks, strongest in mature glutamatergic neurons, involving synaptic, epigenetic, and mitochondrial pathways (74).	Directly supports the fragile-node concept and prioritises cell-type/developmental context.
KMT2A-KDM5C H3K4 balance model	Mouse and neuronal models with transcriptomic, chromatin, dendritic, and behavioural readouts.	Combined perturbation partially corrected dendritic morphology, behavioural traits, transcriptomes, and H3K4me landscapes (66).	Provides experimental support for therapeutic rebalancing along a defined writer-eraser axis.
CHD8 human cerebral organoids	Patient-specific/isogenic cerebral organoids and single-cell transcriptomic profiling.	Altered proliferation and temporally abnormal excitatory and inhibitory neuronal trajectories, with macrocephaly-like organoid enlargement (51).	Connects chromatin remodeling to E/I trajectory imbalance and growth-related neurodevelopmental endophenotypes.
KMT2D/KDM6A Kabuki skeletal growth models	Mouse skeletal phenotyping and RNA-seq of Kmt2d and Kdm6a chondrogenic cell models.	Shared growth deficiency and convergent chondrocyte transcriptomic dysregulation despite distinct genetic etiologies (8, 63)	Shows that convergence can occur at tissue-specific transcriptomic and somatic endophenotype levels.
Electrophysiological and systems-neurology endophenotypes	EEG, sleep physiology, oscillatory dynamics, and clinical epilepsy phenotyping.	EEG slowing, epileptiform discharges, sleep architecture disturbance, and seizure susceptibility (36, 58).	Captures real-time network instability and may monitor whether chromatin load exceeds network capacity.
Neuroimaging network endophenotypes	Structural MRI, diffusion imaging, and functional connectivity approaches.	Cortical volume/thickness, white matter, corpus callosum, cerebellar, hippocampal, and connectivity differences (38, 56).	Provides a systems-level bridge between molecular chromatin perturbation and circuit architecture.

Transcriptomic and cellular model studies further demonstrate that distinct chromatinopathies converge on shared biological pathways. Analyses of autism and related neurodevelopmental disorders have identified dysregulation of synaptic genes, neuronal differentiation programs, activity-dependent transcriptional networks and cell-type-specific developmental trajectories (49, 54). This convergence supports the hypothesis that chromatin regulators act upstream of common network-level processes rather than affecting isolated molecular pathways. Shared dysregulation of synaptic genes, developmental pathways, cytoskeletal programs, ion channel expression, interneuron maturation, or mitochondrial resilience pathways across different chromatinopathies may reveal convergent network effects. Chromatin accessibility assays, such as ATAC-seq or CUT&RUN/CUT&Tag-derived profiling, can further identify which regulatory regions are aberrantly open or closed (55). In principle, integrating these datasets could allow quantification of chromatin load in biologically meaningful domains, such as excitatory neuron differentiation, inhibitory interneuron maturation, cerebellar development, or activity-dependent transcription.

Neuroimaging is the natural systems-level companion to such molecular readouts. Structural MRI can detect cortical thickness differences, volume changes, or white matter abnormalities. Diffusion-based measures can interrogate connectivity. Resting-state fMRI or other network imaging methods may reveal altered large-scale network organisation (56, 57). Although current data are limited and often disorder-specific, a future goal would be to define network endophenotypes that correspond to recurrent chromatin-perturbed developmental states.

Electrophysiology may be especially valuable because it captures real-time network behaviour. EEG slowing, epileptiform discharges, abnormal sleep features, altered oscillatory dynamics, or impaired task-related synchrony may serve as practical indicators of network fragility (58). In some patients, EEG may reveal instability even before clinical seizures emerge. Such findings fit naturally with the concept of a nervous system operating near the boundary of network capacity.

**Figure 2:**
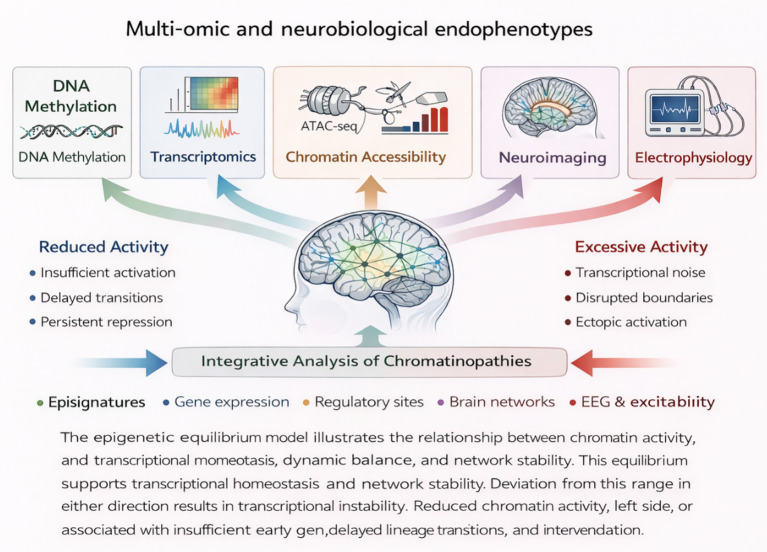
Multi-omic and neurobiological endophenotypes in chromatinopathies. Multi-omic and neurobiological readouts can define measurable endophenotypes of chromatin disequilibrium, DNA methylation, transcriptomic, chromatin accessibility, neuroimaging and electrophysiological data together help identify regulatory direction, vulnerable network domains and disease trajectory.

A mature diagnostic framework would integrate genomic, methylomic, transcriptomic, imaging, and electrophysiological data rather than treat them separately. The goal would not be to generate exhaustive data on every patient, but to use selected endophenotypes to clarify regulatory direction, identify fragile network domains, and monitor trajectory over time (Figure 2).

## 11 X-linked/AR erasers and sex-dependent effects

Chromatin “eraser” enzymes, particularly histone demethylases, are frequently encoded by genes located on the X chromosome, introducing an additional layer of complexity through sex-dependent dosage effects. In contrast to many chromatin “writer” genes, which are often associated with autosomal dominant inheritance and marked dosage sensitivity, eraser genes display more heterogeneous inheritance patterns, including X-linked and autosomal recessive forms. This distinction reflects differences in functional redundancy, buffering capacity, and tolerance to partial loss of function within chromatin regulatory systems. In males, hemizygous loss-of-function variants in X-linked erasers such as *KDM5C* and *KDM6A* typically result in more consistent and severe neurodevelopmental phenotypes, reflecting the absence of a compensatory allele. In females, however, phenotypic expression is often variable due to X-chromosome inactivation. Skewing of X-inactivation can modulate the effective chromatin load at the cellular level, resulting in a spectrum of clinical presentations ranging from asymptomatic carriers to individuals with significant neurodevelopmental impairment.

Within the epigenetic equilibrium framework, these observations can be interpreted as dosage-dependent shifts in chromatin balance. X-linked eraser deficiencies are sensitive to cellular mosaicism: tissue may contain intermingled cell populations with distinct regulatory profiles rather than a uniform shift in chromatin state. This heterogeneity may buffer loss of function in some contexts but may also increase transcriptional variability in neural systems that require synchronised gene expression. Its net effect is therefore likely depend on the balance between cellular buffering and network coherence requirements.

From an evolutionary perspective, the enrichment of chromatin erasers on the X chromosome may represent a mechanism to modulate transcriptional flexibility in a sex-dependent manner. Demethylases often act to fine-tune or reverse transcriptional states, providing a degree of regulatory plasticity. Localisation of these functions on the X chromosome allows differential expression patterns between sexes and introduces mosaic buffering in females, potentially increasing robustness to perturbation while preserving adaptive flexibility.

Clinically, these principles have several implications. First, sex should be explicitly considered in phenotypic interpretation and variant classification. A variant in an X-linked chromatin regulator may have different expected penetrance and expressivity in males and females. Second, skewed X-inactivation may act as a modifier of disease severity and should be investigated where possible, particularly in cases with discordant phenotypes. Third, mosaicism may complicate the interpretation of molecular assays, including methylation signatures or transcriptomic profiling, as averaged signals may obscure cell-specific effects.

Finally, the concept of X-linked buffering reinforces the broader framework of chromatin load and network capacity. It provides a natural example of how biological systems can modulate effective regulatory burden through cellular heterogeneity. Incorporating sex-dependent architecture into models of chromatin equilibrium will therefore be essential for accurate mechanistic understanding and for the development of precision diagnostic and therapeutic strategies.

By contrast, chromatin writer genes, such as *KMT2D*, *CREBBP*, and *EP300,* more commonly exhibit autosomal dominant inheritance, consistent with strong dosage sensitivity and limited buffering capacity. Haploinsufficiency of these writers can shift chromatin equilibrium sufficiently to disrupt transcriptional programs even in the presence of a normal allele. This may explain why writer-related disorders often show high penetrance and relatively consistent phenotypes compared to some eraser-associated conditions.

Importantly, this distinction between writers and erasers is not absolute. Both classes include genes with diverse inheritance patterns, and individual gene function, domain specificity, and developmental context all influence phenotypic outcomes. Nevertheless, the relative enrichment of X-linked and recessive inheritance among erasers supports the concept that chromatin regulatory systems exhibit asymmetric tolerance to perturbation.

**Figure 3:**
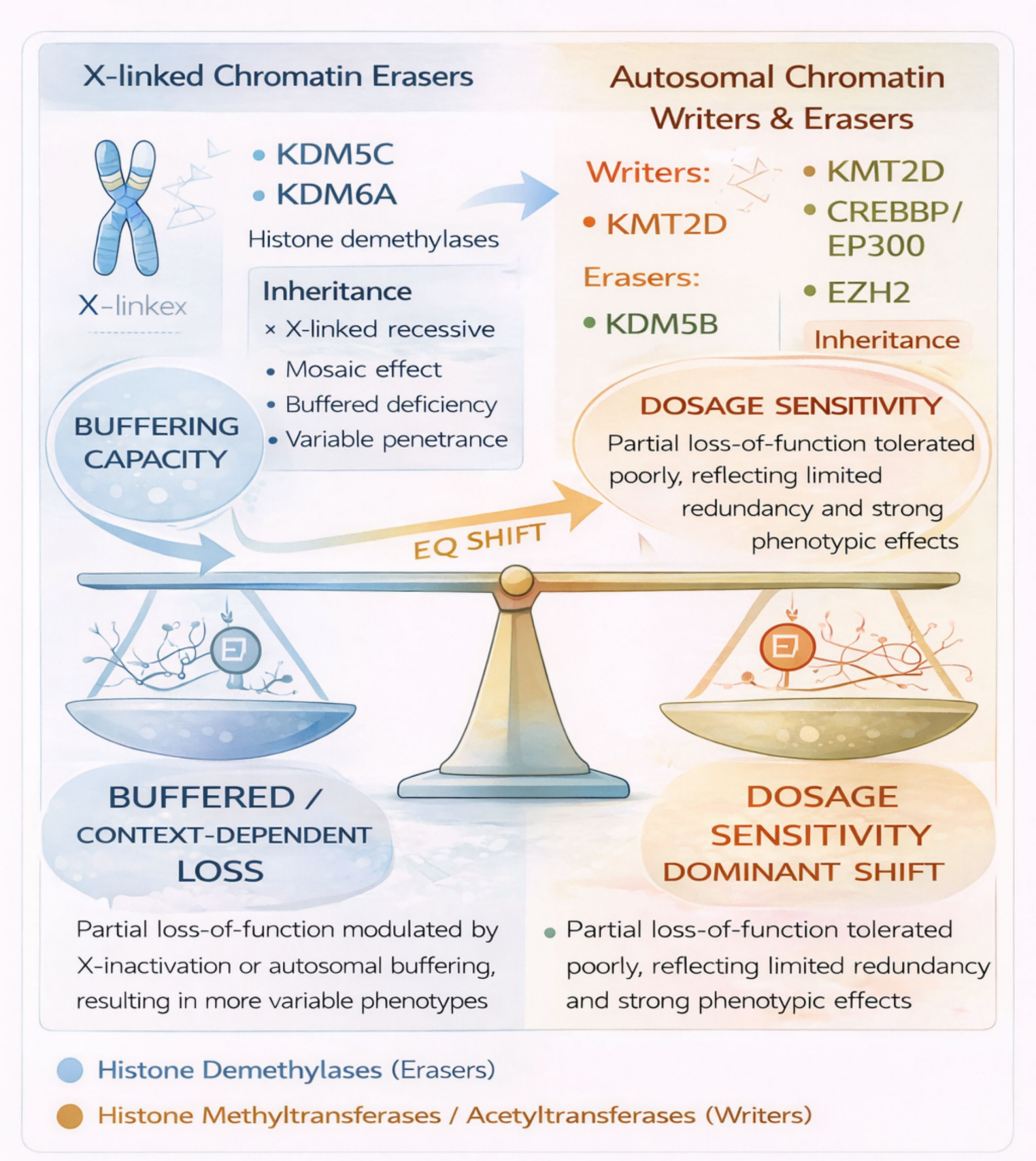
X-linked versus autosomal chromatin regulators and dosage sensitivity. X-linked erasers, autosomal recessive erasers and autosomal dominant writers differ in dosage sensitivity and buffering capacity. These patterns influence effective chromatin load, network capacity, penetrance and phenotypic variability across chromatinopathies.

From a systems perspective, sex-dependent effects in chromatinopathies reflect differences in effective chromatin load and network capacity. In males, absence of redundancy may result in larger deviations from equilibrium, whereas in females, mosaicism may partially buffer these effects but introduce spatial heterogeneity within neural networks. These dynamics provide a mechanistic basis for variable penetrance, sex bias, and phenotypic diversity across chromatin disorders (Figure 3).

## 12 Clinical implications

From a clinical neurology perspective, chromatinopathies are best conceptualised as disorders of network instability arising from dysregulated transcriptional systems. This reframing has important implications for diagnosis, variant interpretation, longitudinal management, and therapeutic strategy. Rather than viewing these conditions as static syndromes defined solely by genetic lesions, they should be understood as dynamic disorders in which molecular perturbation interacts with developmental timing, network architecture, and system-level buffering capacity.

A key implication is the need to move beyond strictly gene-centric diagnostic reasoning. While identification of a pathogenic variant remains central, it is often insufficient to fully explain phenotype or predict clinical trajectory in chromatinopathies. A systems-based approach instead asks how a given variant alters chromatin equilibrium, what direction of regulatory imbalance it implies, and which neural networks are most likely to be affected. This perspective integrates molecular findings with clinical features, including developmental profile, behavioural phenotype, seizure tendency, and neurophysiological markers.

Variant interpretation particularly benefits from this framework. Variants of uncertain significance in chromatin regulators are common, and their classification can be challenging based solely on sequence data. However, when considered alongside systems-level endophenotypes, such as characteristic behavioural profiles, EEG abnormalities, growth patterns, craniofacial gestalt, and DNA methylation episignatures, the overall weight of evidence can be substantially strengthened. Conversely, the absence of network-level coherence may argue against pathogenicity, even in the presence of a rare variant.

The concept of regulatory direction also enables a more nuanced form of diagnostic stratification. Chromatinopathies can be broadly conceptualised as involving insufficient activation-associated function, excessive repression, impaired repression, derepression, or mixed regulatory dysregulation, recognising that these categories are simplifications of a complex continuum. Such stratification may help predict somatic features, identify vulnerable neural domains, and guide future therapeutic decision-making. This approach aligns with mirror endophenotyping, in which opposing molecular perturbations may produce divergent somatic traits but shared neurological outcomes.

Endophenotyping becomes central within this framework. Clinically useful endophenotypes are measurable intermediate features that bridge molecular pathology and clinical presentation. In chromatinopathies, these may include EEG background organisation, epileptiform burden, sleep architecture, cortical connectivity patterns on neuroimaging, language phenotype, motor coordination profile, and disorder-specific methylation signatures. These measures can provide objective markers of network instability and may be particularly valuable in early diagnosis, monitoring progression, and assessing response to intervention.

A further implication is the importance of developmental timing. Chromatin perturbations do not produce static phenotypes; rather, they unfold dynamically across development. Early manifestations such as hypotonia, feeding difficulties, and delayed motor milestones may evolve into language impairment, attentional dysfunction, and behavioural dysregulation, and later into epilepsy or executive dysfunction. Clinical assessment should therefore be longitudinal and adaptive, recognising that phenotype reflects an evolving interaction between chromatin load and network capacity.

The framework also provides a basis for understanding episodic deterioration. Many individuals with chromatinopathies exhibit periods of relative stability punctuated by regression, seizure onset, or behavioural deterioration. These events can be interpreted as shifts in the balance between chromatin load and network capacity, triggered by factors such as illness, sleep disruption, hormonal changes, or environmental stress. This perspective supports proactive management strategies aimed at stabilising network function and preventing decompensation.

## 13 Therapeutic implications

If chromatinopathies are understood as disorders of regulatory imbalance and network fragility, therapeutic goals must be reframed accordingly. The aim is not simply to normalise a mutated protein, but to restore functional equilibrium in a non-linear system. This has several major consequences for therapy design.

The first is that therapeutic success may not require complete correction of the primary molecular defect. In a threshold-based system, reducing chromatin load below the effective capacity of the network may be sufficient to produce meaningful clinical improvement. Partial correction could therefore be highly valuable, especially if delivered during developmentally sensitive periods. This is clinically important because full molecular normalisation may be unrealistic for many chromatin regulators, whereas partial modulation may be pharmacologically or biologically achievable.

The second is that treatment must be dose-sensitive. In linear systems, more correction is usually better. In chromatin systems, overshooting may be harmful. Excessive activation of a repressed pathway or over-inhibition of a compensatory pathway could push the network to the opposite side of equilibrium. Therapies targeting chromatin-modifying enzymes, such as HDAC inhibitors or other epigenetic modulators, therefore require careful titration and biomarker-informed monitoring (59, 60). A drug that modestly improves function in one regulatory context may worsen instability in another.

The third is that timing matters profoundly. Some developmental abnormalities, once embedded in circuit structure, may be only partially reversible. This suggests that early intervention may be essential for therapies aimed at progenitor dynamics, migration, or early synaptogenesis. In contrast, interventions targeting synaptic plasticity, excitability, or network compensation may remain useful later in development (61). The therapeutic landscape is therefore likely to be staged: some approaches are preventative or circuit-building, others modulatory or compensatory.

A fourth implication is that therapies can be directed at different levels of the system. One level is direct chromatin modulation. This includes small molecules that influence histone acetylation, methylation, or associated transcriptional machinery. Such therapies may be particularly useful when a disorder reflects predictable regulatory direction and when systemic toxicity can be managed. The promise of this approach lies in its capacity to influence broad transcriptional states, but that same breadth is also its major risk.

A second level is locus-specific epigenome editing. CRISPR-based transcriptional and epigenetic editing strategies raise the possibility of modulating selected enhancers or promoters without globally altering chromatin. For chromatinopathies, this could be especially appealing if a limited number of fragile network hubs account for much of the phenotype. By restoring expression at these nodes, it may be possible to improve function without rebalancing the entire epigenome. However, identifying the correct targets, developmental windows, and delivery systems remains a substantial challenge (60).

A third therapeutic level is downstream network modulation. If chromatin imbalance ultimately converges on synaptic dysfunction, excitability imbalance, or impaired plasticity, then therapies acting at these downstream levels may still be highly effective. Antiseizure medications are an obvious example, but the broader principle includes modulation of inhibitory-excitatory balance, enhancement of synaptic stability, support of sleep architecture, reduction of neuroinflammation, or optimisation of metabolic resilience (48). In this view, even conventional neurological treatments can be understood as reducing effective chromatin load or increasing network capacity.

A fourth level is the enhancement of network capacity. This is a particularly useful concept because it reframes treatment away from exclusive focus on primary defect correction. Network capacity may be increased through mechanisms that improve synaptic plasticity, neuronal metabolic support, myelination, sleep quality, or behavioural regulation. Early developmental therapies, language intervention, structured educational support, seizure control, sleep optimisation, treatment of anxiety, and management of sensory dysregulation may all increase the brain’s ability to compensate. This does not trivialise supportive care; rather, it situates it within a mechanistic framework of resilience enhancement.

The concept of combination therapy follows naturally. Because chromatinopathies are systems-level disorders, single-agent intervention is unlikely to be universally sufficient. A rational therapeutic program may combine one agent that nudges chromatin state toward equilibrium, another that stabilises excitability, and a third that supports plasticity or behavioural adaptation. The optimal combination would likely vary by developmental stage and clinical phenotype.

This framework also changes how clinical trials should be designed. Outcome measures should not be limited to global cognition or seizure frequency alone, but should include biomarkers of regulatory state and network function. EEG measures, sleep quality, language trajectories, methylation signatures, connectivity markers, or composite developmental endophenotypes may be more sensitive to biological effect than blunt endpoint scales. Trials should also consider stratifying patients by predicted regulatory direction or endophenotypic profile, not simply by gene label.

**Figure 4:**
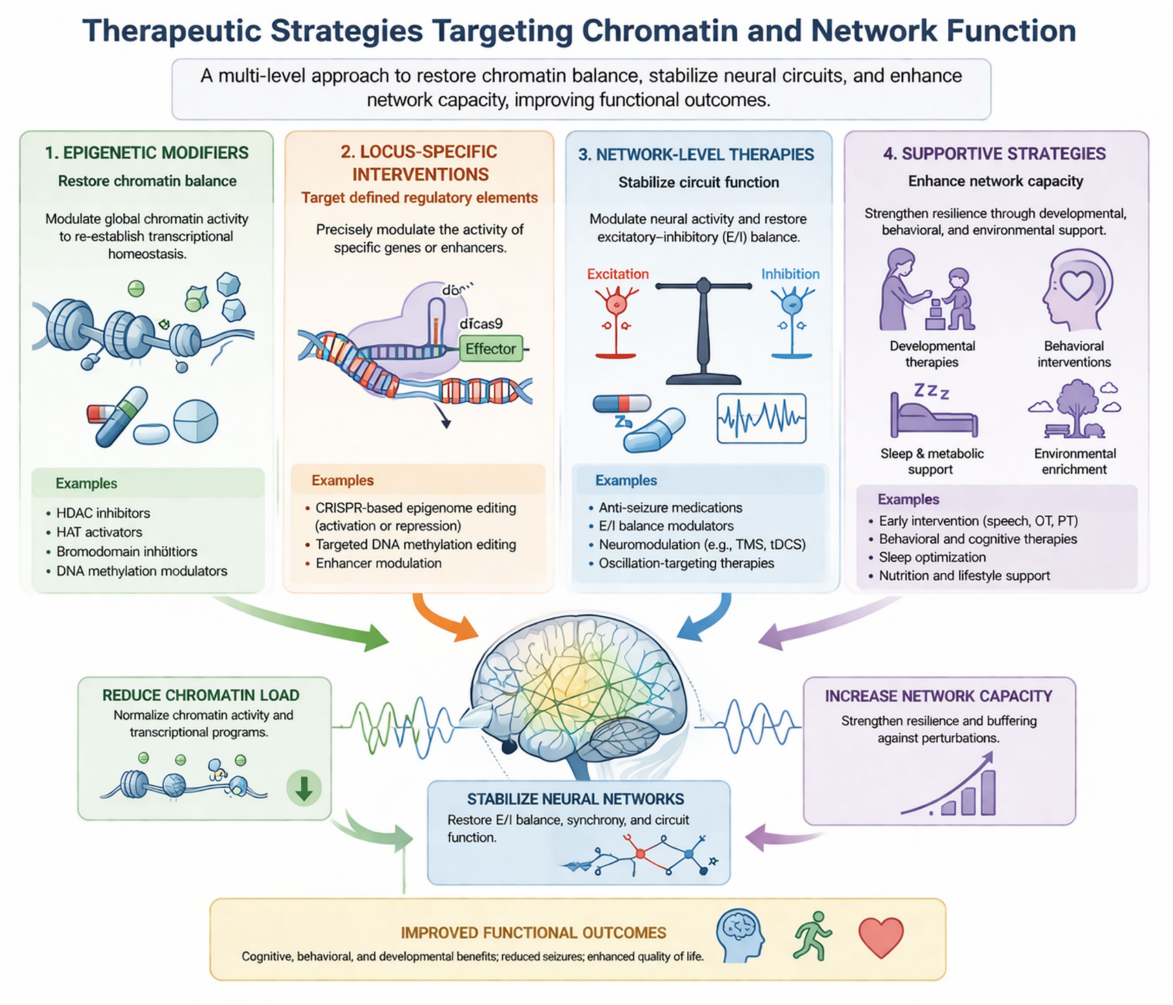
Therapeutic strategies targeting chromatin and network function. Therapeutic strategies can target chromatin regulation, locus-specific epigenetic control, downstream circuit instability or network capacity. These complementary approaches aim to restore regulatory balance, reduce effective chromatin load, and stabilise neural networks.

Finally, therapeutic optimism must be tempered by realism. Chromatin regulation is fundamental to biology, and systemic intervention carries genuine risk. The goal is not indiscriminate manipulation of the epigenome, but carefully informed modulation based on understanding of equilibrium, load, fragility, and timing. The deeper the biological model, the safer and more rational the intervention becomes (Figure 4).

## 14 Future directions and conceptual priorities

Several priorities emerge from this framework. First, there is a need to better define fragile network hubs across chromatinopathies. Which genes, pathways, or circuit motifs most consistently mediate phenotypic convergence? Second, quantitative models of chromatin load need to be developed. This will require integration of multi-omic and neurophysiological data rather than reliance on single biomarkers. Third, developmental timing must be incorporated more explicitly into both mechanistic studies and therapy design. Fourth, diagnostic frameworks should increasingly combine genetic findings with methylation, electrophysiology, and imaging endophenotypes. Fifth, more work is needed on sex-dependent modifiers, mosaicism, and background resilience factors that alter network capacity.

A further conceptual priority is to distinguish between reversible and irreversible consequences of chromatin imbalance. Some phenotypes may reflect fixed developmental misspecification, whereas others may arise from ongoing network instability and therefore remain modifiable. This distinction is central to therapeutic strategy. Another important area is the interface between chromatinopathies and more common neurodevelopmental and psychiatric disorders. The same fragile network principles identified in Mendelian disorders may illuminate broader dimensions of autism, epilepsy, intellectual disability, or even mood and psychotic disorders, especially where regulatory burden is polygenic rather than monogenic.

## 15 Conclusion

Chromatinopathies provide a unique window into the principles governing gene regulation in human neurodevelopment. Despite molecular diversity, these disorders converge on a recognisable neurological phenotype, suggesting shared vulnerability at the level of gene regulatory networks and neural circuitry.

The epigenetic equilibrium model offers a unifying framework for understanding this convergence. By proposing that neurodevelopment depends on maintaining chromatin states within a constrained functional range, it explains why deviations in either direction can destabilise transcriptional homeostasis and produce similar neurological outcomes.

The related concepts of chromatin load, network capacity, and chromatin network fragility extend this framework into a systems-level model that links molecular perturbation to circuit dysfunction, clinical variability, and therapeutic opportunity. Integration of multi-omic and neurobiological endophenotypes further enables translation of this model into diagnostic practice.

This perspective supports a shift from gene-centric to systems-based reasoning in neurodevelopmental disorders. It suggests that therapeutic strategies should aim to restore regulatory balance, reduce effective load, or enhance network capacity rather than focus exclusively on complete molecular correction.

**Figure 5:**
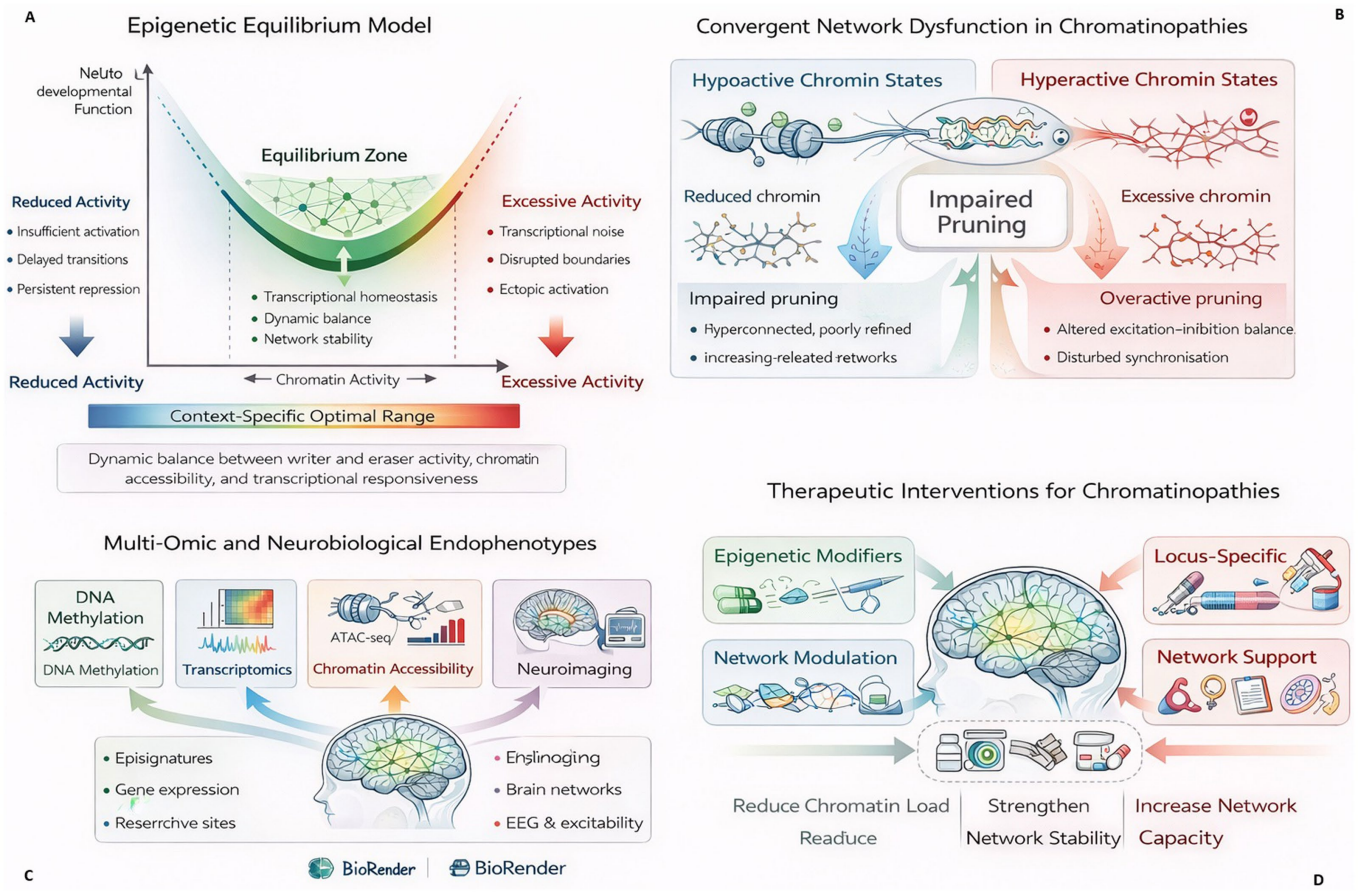
Epigenetic equilibrium, network convergence, endophenotypes, and therapeutic strategies in chromatinopathies. The figure summarises four linked components of the model: **(A)** context-specific epigenetic equilibrium, **(B)** convergent network dysfunction and pruning abnormalities, **(C)** multi-omic and neurobiological endophenotyping, and **(D)** therapeutic strategies that restore balance, reduce chromatin load, stabilise circuits, or increase network capacity.

Ultimately, chromatinopathies should not be viewed as a heterogeneous collection of rare syndromes, but as a coherent class of disorders that reveal fundamental principles of transcriptional regulation and network stability in the developing human brain (Figure 5).
